# Phase-Dependent Crossed Inhibition Mediating Coordination of Anti-phase Bilateral Rhythmic Movement: A Mini Review

**DOI:** 10.3389/fnhum.2021.668442

**Published:** 2021-05-06

**Authors:** Koichi Hiraoka

**Affiliations:** College of Health and Human Sciences, Osaka Prefecture University, Habikino, Japan

**Keywords:** rhythmic movement, bilateral coordination, locomotion, central pattern generator, extensor half center, flexor half center, half center model, crossed inhibition

## Abstract

The activity of the left and right central pattern generators (CPGs) is efficiently coordinated during locomotion. To achieve this coordination, the interplay between the CPG controlling one leg and that controlling another must be present. Previous findings in aquatic vertebrates and mammalians suggest that the alternate activation of the left and right CPGs is mediated by the commissural interneurons crossing the midline of the spinal cord. Especially, V0 commissural interneurons mediate crossed inhibition during the alternative activity of the left and right CPGs. Even in humans, phase-dependent modulation of the crossed afferent inhibition during gait has been reported. Based on those previous findings, crossed inhibition of the CPG in one leg side caused by the activation of the contralateral CPG is a possible mechanism underlying the coordination of the anti-phase rhythmic movement of the legs. It has been hypothesized that the activity of the flexor half center in the CPG inhibits the contralateral flexor half center, but crossed inhibition of the extensor half center is not present because of the existence of the double limb support during gait. Nevertheless, previous findings on the phase-dependent crossed inhibition during anti-phase bilateral movement of the legs are not in line with this hypothesis. For example, extensor activity caused crossed inhibition of the flexor half center during bilateral cycling of the legs. In another study, the ankle extensor was inhibited at the period switching from extension to flexion during anti-phase rhythmic movement of the ankles. In this review article, I provide a critical discussion about crossed inhibition mediating the coordination of the anti-phase bilateral rhythmic movement of the legs.

## Introduction

Anti-phase bilateral rhythmic movement is produced during locomotion in mammals. The central pattern generators (CPGs) produce the rhythmic movement of the limbs (Guertin, 2009). Previous studies using a split belt treadmill suggested that the CPG in each side produces rhythmic movement of the ipsilateral leg during locomotion (Dietz et al., 1994; Prokop et al., 1995; Yang et al., 2005; Choi and Bastian, 2007). An experiment on animals has shown that V0 commissural interneurons crossing the midline of the spinal cord play a role in the left-right alternate activity of the locomotion (Talpalar et al., 2013). Those interneurons contribute to crossed inhibition of the contralateral motor output (Rybak et al., 2015; Danner et al., 2017). Even in humans, crossed afferent inhibition is present (Stubbs and Mrachacz-Kersting, 2009; Stubbs et al., 2011a), and is modulated during gait (Stubbs et al., 2011b; Hanna-Boutros et al., 2014). There are several important findings indicating that the phase-dependent crossed inhibition occurs during the anti-phase bilateral movement in humans (Ting et al., 1998; Hiraoka et al., 2014). In this review, I introduce important previous findings on this issue, and discuss crossed inhibition mediating the coordination of the anti-phase rhythmic movement of the legs.

### Left and Right CPGs

The CPGs in the spinal cord produce locomotion in mammals (Brown, 1911, 1914; Frigon, 2012). Even in humans, the existence of the CPG in the spinal cord has been suggested by a previous finding in patients with complete spinal cord injury (Dimitrijevic et al., 1998). The activity of the CPG is explained by the half center model in which the flexor and extensor half centers are alternatively activated during rhythmic movement (McCrea and Rybak, 2008; Guertin, 2009).

In rats, locomotor activity in each side of the limb was produced even the spinal cord was separated with a midline cut (Kudo and Yamada, 1987). This finding indicates that the neural networks responsible for producing rhythmic movement of the leg (i.e., CPG) are located on each side of the spinal cord. Even in humans, this view seems to be true, according to previous findings that different patterns of the stepping movements are produced in the legs when humans gait over the split-belt treadmill in which each of the two belts under each leg runs at a different speed or direction. For example, the stance phase of one leg on the faster moving belt was shortened relative to the contralateral leg on the slower moving belt (Dietz et al., 1994; Prokop et al., 1995). Moreover, even in infant humans, the extra step was produced for the leg on the faster moving belt (Yang et al., 2005). In addition, when the split belts ran in the opposite directions, the infants produced forward stepping in one leg and backward stepping in the contralateral leg. Such opposite stepping between the legs during the opposite running of the moving belts has been observed in adult humans as well (Choi and Bastian, 2007).

### Crossed Pathways

Even though the CPG on each side of the spinal cord is possible to produce rhythmic movement in each leg separately, activity of the left and right CPGs must be coordinated during locomotion in humans, according to an observation that humans move the legs coordinately during gait (Perry, 1992). Previous studies in mammals have reported the existence of the pathways crossing the midline of the spinal cord ventral commissure, namely commissural interneurons, and those neurons mediate the coordination of the left and right motor activities (Kjaerulff and Kiehn, 1996, 1997; Butt et al., 2002; Kiehn, 2006; Goulding, 2009). In some aquatic vertebrates, there are three populations of the commissural interneurons; class 1, 2, and 3 (Butt et al., 2002). Importantly, the class 1 population of the commissural interneurons inhibits the contralateral motor output of the CPG. In the other words, the class 1 population of the commissural interneurons, acting for crossed inhibition, plays a role in the coordination of the alternate activity of the left and right motor systems at least in those animals.

V0–V3 commissural interneurons represent the classes of the postmitotic spinal interneurons based on the transcription factor expression (Butt et al., 2002; Kiehn et al., 2010). Especially, V0 interneurons play a role in the left-right alternation of the motor output during locomotion (Talpalar et al., 2013). V0 commissural interneurons are the major class of neurons in the ventral spinal cord. There are subdivisions of the V0 commissural interneurons; excitatory V0_V_ interneurons, and inhibitory V0_D_ interneurons. The role of the V0 commissural interneurons on the left-right alternation of the motor output is dependent on the frequency of the locomotion cycle (Talpalar et al., 2013; Bellardita and Kiehn, 2015). Ablation of the excitatory V0_V_ interneurons maintained an alternate activity at low-frequency cycle of locomotion, but switched to a synchronized activity at high-frequency cycle of locomotion (Talpalar et al., 2013). Ablation of the V0v commissural interneurons eliminated expression of the trot, but the walk was still present (Bellardita and Kiehn, 2015). The cycle frequency of the trot was faster than that of the walk (Bellardita and Kiehn, 2015). Thus, the findings indicate that V0_V_ commissural interneurons play a role in the left-right alternation during the high-frequency cycle of the locomotion. In contrast, ablation of the inhibitory V0_D_ interneurons eliminated the left-right alternation at low-frequency cycle of the locomotion, but maintained it at high-frequency cycle of the locomotion (Talpalar et al., 2013). Thus, the inhibitory V0_D_ interneurons play a role in the low-left-right coordination of the low-frequency locomotion.

**Figure 1:**
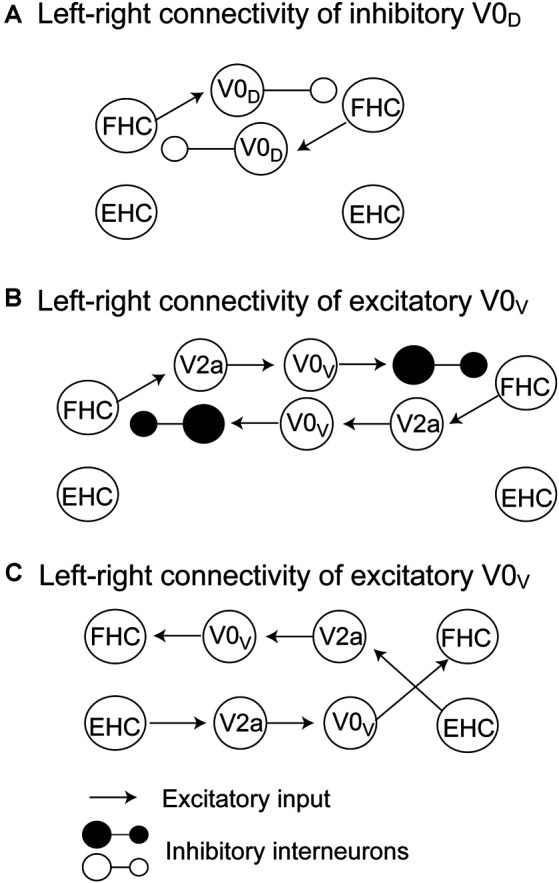
The pathways mediating crossed inhibition between the left and right half centers proposed by Rybak et al. (2015) and Danner et al. (2017). The crossed inhibitory pathway involving V0_D_ commissural interneurons is shown in panel **(A)**. The crossed inhibitory pathways involving V0_V_ commissural interneurons are shown in panels **(B)** and **(C)**. The detailed explanation is presented in the text. FHC, flexor half center; EHC, extensor half center.

The pathways mediating the alternate activity of the half centers during locomotion have been proposed by a computational model (Rybak et al., 2015; Danner et al., 2017). On the one hand, inhibitory V0_D_ interneurons receive exaction from the ipsilateral flexor centers and inhibit the contralateral flexor half center (Figure 1A). On the other hand, V0_V_ commissural interneurons are involved in two possible connections. One is that the excitatory V2a interneurons receive excitation from the ipsilateral flexor center and excite V0_V_ interneurons but inhibit contralateral flexor half center through the inhibitory interneurons (Figure 1B). Another is that the excitatory V2a interneurons receive excitation from the ipsilateral extensor half center and excite V0_V_ interneurons, and then, V0_V_ interneurons excite the contralateral flexor half center (Figure 1C). Taken together, crossed inhibition mediated by the V0 commissural interneurons plays a role in the crossed inhibition mediating the left-right alternate activity of the half centers.

### Crossed Inhibition During Unilateral Movement

According to the discussion above, the alternate activity of the left and right CPGs is likely mediated by crossed inhibition between the CPGs; activity of the half center on one side inhibits the contralateral half center. Crossed inhibition induced by the activity of the CPG in one leg has been examined by observing the effect of the rhythmic movement of one leg on the soleus H-reflex in the contralateral leg at rest in humans. The soleus H-reflex at rest was suppressed by the contralateral leg cycling in the flexion phase and the end of the extension phase (McIllroy et al., 1992). In another study by Mori and colleagues, active rhythmic movement of the ankle caused tonic suppression of the contralateral soleus H-reflex at rest throughout the whole movement phases (Mori et al., 2015). Those findings indicate that the unilateral activation of the CPG on one leg side induces suppression of the H-reflex pathway in the contralateral ankle extensor.

However, those previous findings on the unilateral rhythmic movement are not direct evidences indicating the mechanism underlying the coordination of the leg movements during the anti-phase bilateral rhythmic movement during which both left and right half centers are alternately activated. In addition, we have to note supraspinal influence on crossed inhibition during unilateral movement. Unimanual activation of one finger muscle increases interhemispheric inhibition from the active hemisphere to the resting hemisphere (Vercauteren et al., 2008). This interhemispheric inhibition is to prevent unwanted motor output of the limb at rest (mirror activity) during the activity of the contralateral limb (Vercauteren et al., 2008). Such interhemispheric inhibition may be a possible mechanism underlying the unilateral rhythmic movement-induced suppression of the contralateral soleus H-reflex.

### Crossed Afferent Inhibition

In humans, one important neural event indicating the existence of the inhibitory pathways crossing the midline of the spinal cord is crossed afferent inhibition. The soleus H-reflex in the leg at rest was suppressed by the conditioning stimulation of the tibial nerve in the contralateral leg given 3–33 ms after the test stimulus (Stubbs et al., 2011a). This finding indicates that the activation of the pathways crossing the midline of the spinal cord induced by the afferent input suppresses the contralateral soleus H-reflex pathway. The central delay of this crossed inhibition was 3 ms, indicating that crossed inhibition is mediated by the crossed pathway in the spinal cord, and the commissural interneurons are likely involved in this process (Hanna-Boutros et al., 2014). Moreover, crossed inhibition was modulated by transcranial magnetic stimulation over the primary motor cortex, indicating that the pathways mediating the crossed afferent inhibition receive descending input (Hanna-Boutros et al., 2014).

Soleus H-reflex amplitude is suppressed by the contralateral cutaneous stimulation of the dorsum of the foot in the early stance phase of gait (Suzuki et al., 2014). The late stance phase of the soleus muscle activity is inhibited by electrical stimulation to the contralateral leg in the late phase of the swing leg (Stubbs et al., 2011b). Group II crossed afferent inhibition was reduced especially in the stance phase of the gait cycle (Hanna-Boutros et al., 2014). Those findings indicate that the crossed afferent inhibition is phase-dependently modulated during gait.

Such crossed afferent inhibition is possible to be induced by passive movement of the contralateral leg. For example, passive rhythmic movement of one leg suppressed the contralateral soleus H-reflex at rest (McIllroy et al., 1992; Collins et al., 1993; Cheng et al., 1998; Misiaszek et al., 1998). Either in-phase or anti-phase rhythmic passive movement of both hips or unilateral rhythmic passive movement of the hip contralateral to the tested side suppressed the soleus H-reflex (Stanislaus et al., 2010). During passive movement, the descending motor drive is absent, but the somatosensation induced by the movement is present. Thus, suppression of the soleus H-reflex induced by the passive movement of the contralateral leg is likely mediated by the afferent discharge induced by the somatosensation. This means that the suppression of the soleus H-reflex induced by the passive rhythmic movement of the contralateral leg reflects the crossed afferent inhibition.

Based on those findings, the activity of one half center is inhibited not only by the active rhythmic movement of the contralateral leg, but also by the passive rhythmic movement of that. The somatosensation is induced, but the CPG is inactive during passive movement. It has been stated that the CPG is capable of reorganizing the sensory input to reconfigure itself to evoke the appropriate pattern (Frigon, 2012). Accordingly, one hypothetical explanation for the crossed afferent inhibition is that the activity of the CPG in one leg side induces afferent discharge, and this inhibits the contralateral half center. One previous finding against this hypothesis is that the interlimb coordination of the air stepping in cats was not changed by sensory perturbations (Giuliani and Smith, 1985). In future studies, the hypothesis must be tested in humans. Investigation of the crossed inhibition without somatosensation may be possible if one uses ischemic nerve block (Hayashi et al., 2019) or local anesthesia. Further studies on the patients with diabetic polyneuropathy in which the proprioception is selectively impaired (Bloem et al., 2000) may also be a nice idea for investigating crossed inhibition without somatosensation.

### Crossed Inhibition During Bilateral Movement

**Figure 2:**
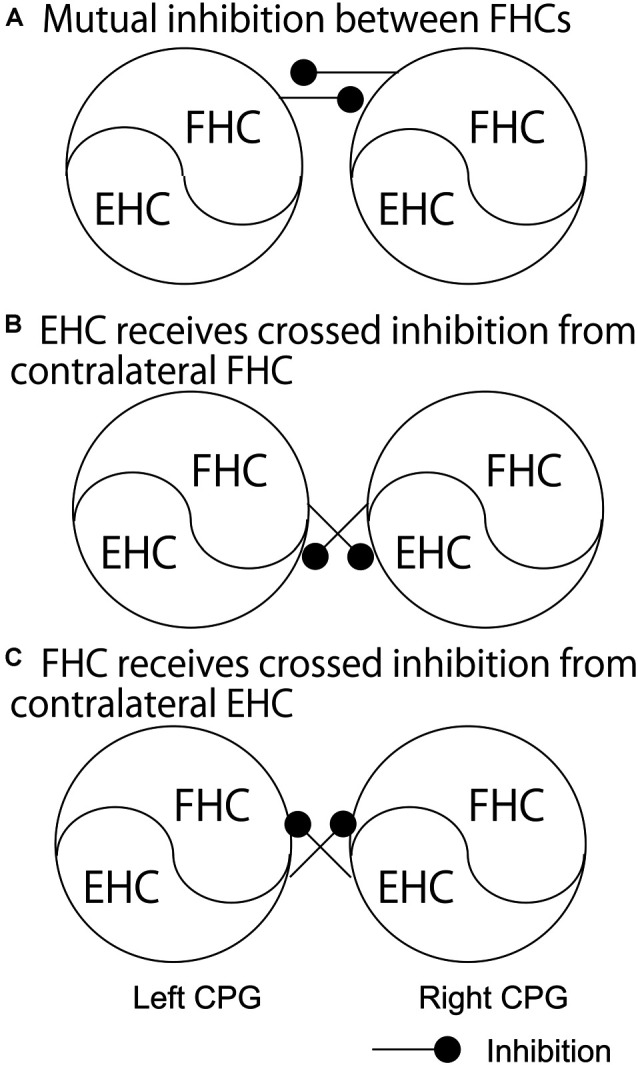
The pathways regarding the phase-dependent crossed inhibition between the left and right half centers. A model proposed by Dietz **(A)**, a model indicated by the finding in the study by Frigon and colleagues **(B)**, and a model proposed by Ting and colleagues **(C)** are shown. Those pathways are discussed in the text. FHC, flexor half center; EHC, extensor half center.

As discussed above, crossed inhibition of the contralateral CPG is a possible mechanism underlying the anti-phase bilateral rhythmic movement. Crossed inhibition is supposed to be achieved through mutual inhibition between the extensor half centers, between the flexor half centers, or between the extensor half center and contralateral flexor half center (see “Crossed Pathways” section and Figure 1). As shown in Figure 2A, Dietz (2002) hypothesized that the neural circuits coordinating the leg flexor activity of both sides during the swing phase of locomotion (i.e., flexor half center) mutually inhibit one another (Dietz, 2002). By contrast, the extensor half-centers on each side have no crossed inhibitory connections, agreeing with the coexistence of the stance phase on the two sides.

This hypothesis by Dietz was supported by a previous finding on the split-treadmill gait (Yang et al., 2005). When infants walk on the split-belt treadmill with different speeds of belts under the legs, coactivation of the left and right extensors was greater than that of the left and right flexors. Accordingly, the authors of this study speculated that the mutual inhibition between the left and right flexor half centers is greater, compared to the extensor half centers. Moreover, the hypothesis by Dietz is in line with the computational model of the V0_D_ commissure interneurons proposed by Rybak et al. and Danner et al. (Rybak et al., 2015; Danner et al., 2017; see “Crossed Pathways” section and Figure 1 in the present review), shown in Figure 1A. In contrast, short-latency crossed inhibitory response, followed by the long-latency excitatory response, on the extensors was observed in the stance phase during locomotion in cat (Frigon and Rossignol, 2008). This indicates that the extensor half center in the stance phase side of the limb receives crossed inhibition from the contralateral flexor half center during locomotion (Figure 2B). This finding conflicts with the hypothesis by Dietz.

### Phase-Dependent Crossed Inhibition During Bilateral Movement

In human experiments, crossed inhibition of the CPG during activity of the contralateral CPG has been examined by comparing the motor output between the unilateral and bilateral rhythmic movements. For example, peak power during unilateral cycling was greater than that during the anti-phase bilateral cycling (Dunstheimer et al., 2001). This finding indicates that the activity level of the rhythmically moving one leg is less when the contralateral side moves rhythmically with an anti-phase fashion.

Previous findings on the phase-dependent inhibition of the rhythmically moving one leg induced by the rhythmic movement of the contralateral leg were not in line with the hypothesis proposed by Dietz. For example, during bilateral anti-phase cycling, the activity of the tibialis anterior, biceps femoris, and rectus femoris muscles in the flexion phase was less than that during the unilateral cycling of the tested leg (Ting et al., 1998). This finding indicates that the activation of the extensors in the extension phase of one leg decreases the activity of the contralateral leg muscles in the flexion phase in which the flexor half center is active. Based on this finding, Ting and colleagues hypothesized that the activity of the extensor half center inhibits the activity of the contralateral flexor half center (Figure 2C). This view is in line with the proposed model regarding crossed inhibition mediated by V0_V_ commissural interneurons (Figure 1C). However, this view may not be applicable for the explanation of the anti-phase bilateral coordination. That is, the activation of the extensor half center inhibits the contralateral flexor half center which is active at this moment during the anti-phase bilateral movement based on this view, but this is not functionally meaningful for the anti-phase bilateral movement.

**Figure 3:**
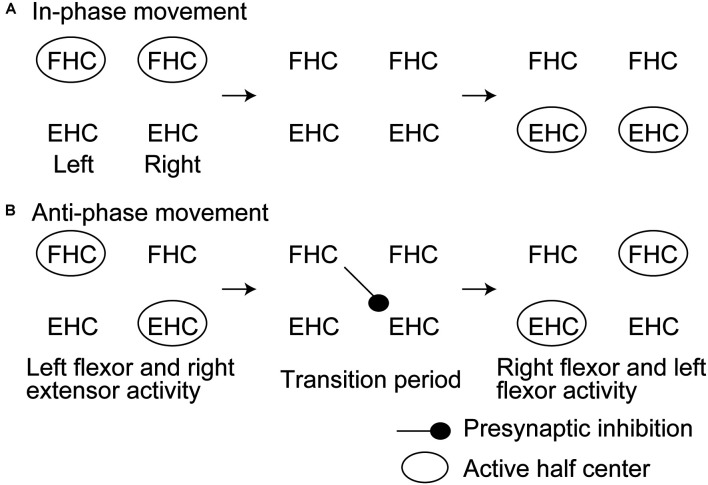
Hypothesized time course of the half center activities and crossed inhibition during in-phase **(A)** and anti-phase bilateral rhythmic ankle movement **(B)** proposed by Hiraoka and colleagues. This model is discussed in the text. FHC, flexor half center; EHC, extensor half center.

Another previous study had tested the soleus H-reflex of the rhythmically moving ankle with and without rhythmic movement of the contralateral ankle (Hiraoka et al., 2014). The soleus H-reflex during the anti-phase bilateral ankle movement at the period switching the movement from the extension to the flexion of the tested ankle while switching the contralateral ankle movement from the flexion to the extension was smaller than that at the same period of the tested ankle during the unilateral movement of the tested ankle. This finding supports a view that crossed inhibition of the extensor half center is produced at the period switching the activity from the extensor to flexor half center while the contralateral side is at the period switching the activity from the flexor to extensor half center (Figure 3). A previous study reported that the activity of the bifunctional thigh muscle increased at the transition period between the extension and flexion of bilateral anti-phase cycling (Kautz et al., 2002). The authors of this previous study speculated that the mutual modulation of the left and right motor systems in the legs is produced at the switching phase between the flexion and extension to ensure the smooth switching of the flexor and extensor half center activity. The finding by Hiraoka and colleagues mentioned above supports this view.

A previous study reported that the soleus H-reflex during bilateral leg cycling was not significantly different from that during the unilateral cycling of the leg contralateral to the tested side (McIllroy et al., 1992). This finding was inconsistent with the previous finding by Hiraoka and colleagues. In the study by McIlroy and colleagues, both ankles were fixed, but the hip and knee were moved during the cycling movement. In contrast, in the study by Hiraoka and colleagues, the ankle, in which the soleus muscle was the prime mover of the extension movement, was actively moved. Based on this, those conflicting findings indicate that crossed inhibition targeting the different segmental levels of the spinal cord is different from the inhibition targeting the same segmental level of the spinal cord. Accordingly, phase-dependent suppression of the soleus H-reflex during the anti-phase bilateral movement of the ankles observed in the study by Hiraoka and colleagues is well explained by a view that crossed inhibition of the soleus H-reflex during the anti-phase bilateral movement occurs within the intrasegmental level of the spinal cord.

An important finding in the previous study by Hiraoka and colleagues is that such phase-dependent inhibition was not present during the in-phase movement of the ankles. This finding suggests that crossed inhibition during the switching period of bilateral rhythmic movement is particularly present during the anti-phase bilateral movement. It has been suggested that the control process of the in-phase bilateral movement of the hands is different from that of the anti-phase movement (Shih et al., 2019). As shown in previous studies on the aquatic vertebrates, the population of the pathways crossing the midline of the spine to mediating the synchronous motor activity of the left and right motor systems is different from that mediating the alternate motor output of those (Butt et al., 2002). There is a hypothetical view that the inhibitory commissural interneurons are active during the alternate activity of the left and right motor systems during walking whereas the excitatory commissural interneurons are active during the synchronous activity of those during galloping or hopping (Kiehn, 2006). More recently, it has been reported that crossed inhibition between the left and right half centers mediated by V0 or V2a commissural interneurons particularly contributes to the left-right alternation of the motor output during locomotion in animals (Crone et al., 2009; Talpalar et al., 2013; Bellardita and Kiehn, 2015). According to those previous findings, phase-dependent crossed inhibition particularly during the anti-phase bilateral movement observed in the study by Hiraoka and colleagues may reflect a fact that crossed inhibition is mediated by such class-specific activity of the commissural interneurons.

### Presynaptic Origin of Crossed Inhibition

Because pre-stimulus background muscle activity level was equivalent between the conditions in this previous study by Hiraoka and colleagues, suppression of the soleus H-reflex during bilateral rhythmic movement observed is likely presynaptic origin. In humans, rhythmic leg activation during gait was greatly controlled by presynaptic inhibition (Stein and Capaday, 1988; Stein, 1995). The force at the toe contact of the stance phase influenced the presynaptic inhibition of the contralateral limb during locomotion in rat (Hayes et al., 2012). Crossed inhibition during locomotion was induced without change in the electromyographic activity, indicating that crossed inhibition is presynaptic origin (Suzuki et al., 2014). Those previous findings indirectly support a view that crossed inhibition at the switching period of the extensor and flexor half center activities during the anti-phase bilateral movement observed in the previous study by Hiraoka and colleagues is mediated by the presynaptic inhibition. Some commissural interneurons directly project to the contralateral motoneurons, but others provide indirect influence on the motoneurons through the inhibitory interneurons (Kiehn et al., 2010). The presynaptic origin of the phase-dependent crossed inhibition found in the study by Hiraoka and colleagues may be related to this indirect influence mediated by the pathway that involves the commissural interneurons.

The conflict between the finding by Hiraoka and colleagues and the finding by Ting and colleagues may be due to the difference in the measurement of the motor status. The experiment by Ting and colleagues observed the EMG and force. Thus, the finding indicates the direct inhibition of the motoneurons causing the decrease in the motor output. In contrast, the experiment by Hiraoka and colleagues observed the suppression of the H-reflex with equal EMG activity. Thus, the finding in the study by Hiraoka and colleagues indicates the inhibition of the excitatory synaptic transmission to the motoneurons. This process is not direct inhibition of the motoneurons, and thus, does not indicate the change in the motor output. Such difference may be related to the conflicting findings between the studies.

### Remained Issues on Phase-Dependent Crossed Inhibition

In the studies on bilateral coordination in humans, as introduced in the present study, actual locomotion is not performed; pedaling movement (Ting et al., 1998) or ankle movement (Hiraoka et al., 2014) was performed. Thus, the findings in those studies are not direct evidences indicating crossed inhibition during locomotion. In future studies, crossed inhibition during actual locomotion (i.e., gait) must be tested. Studies using a split-belt treadmill, as investigated to observe the CPGs in previous studies (Dietz et al., 1994; Prokop et al., 1995; Yang et al., 2005; Choi and Bastian, 2007), might be useful to investigate this issue. Another issue that remains to be investigated is whether such crossed inhibition actually contributes to bilateral coordination. The decrease in the EMG activity, force, or H-reflex excitability does not indicate the change in bilateral coordination. Coordination of the two limbs has been studied through observing stability, accuracy, or transition of the relative phase between the two-limb movements (Kelso and Schöner, 1988; Abe et al., 2003; Asai et al., 2003; Nomura et al., 2016). Such measurements are the direct evidence indicating bilateral coordination. Thus, further studies observing those measurements with testing crossed inhibition must be conducted to test a view that crossed inhibition contributes to bilateral coordination in humans.

### Descending Motor Drive

The mesencephalic locomotor region and the lateral hypothalamus project to the reticulospinal neurons, and those neurons project to the spinal cord for activating the CPGs (Jordan et al., 2008; Frigon, 2012). Accordingly, the CPGs are under the control of the descending pathways. At least in the upper extremities, the supraspinal pathways crossing the midline are likely involved in bilateral coordination (Carson, 2005). Selective ablation of the descending spinal neurons perturbed interlimb coordination during high-speed stepping in mice (Ruder et al., 2016). In patients with Parkinson’s disease, bilateral coordination was impaired (Abe et al., 2003; Asai et al., 2003). Crossed inhibition was modulated by transcranial magnetic stimulation over the primary motor cortex (Hanna-Boutros et al., 2014). Based on those previous findings, supraspinal descending motor drive is not ruled out from the mechanism underlying the left-right coordination of the rhythmic bilateral movement. To rule out the possible effect of the supraspinal descending motor drive on bilateral coordination, further studies on the patients with complete spinal cord injury, as investigated previously for observing the activity of the CPG in humans (Dimitrijevic et al., 1998), are needed.

## Conclusions

In this article, previous studies on the phase-dependent crossed inhibition underlying the anti-phase coordination of the rhythmic movement of the legs were reviewed. V0 commissural interneurons mediating crossed inhibition of the contralateral half center likely contribute to the alternate activity of the left and right CPGs. Crossed inhibition of the contralateral leg motor system has been reported in previous studies in humans. Accordingly, alternate activity between the left and right CPGs may be coordinated by crossed inhibition of the spinal cord in humans. On the one hand, a previous finding suggested that the flexor half center is inhibited during the activity of the contralateral extensor half center. On the other hand, another recent study indicated that the extensor half center is inhibited at the period switching the activity from the extensor to flexor half center while the contralateral limb is at the period switching the activity from the flexor to extensor half center. Future studies are needed for further insight into those findings.

## Author Contributions

KH wrote this article.

## Conflict of Interest

The author declares that the research was conducted in the absence of any commercial or financial relationships that could be construed as a potential conflict of interest.
